# Supplementary material for: Meropenem Pharmacokinetics and Target Attainment in Critically Ill Patients Are Not Affected by Extracorporeal Membrane Oxygenation: A Matched Cohort Analysis
Source: Microorganisms. 2021 Jun 16;9(6):1310. doi: 10.3390/microorganisms9061310 (PMC8234236; doi:10.3390/microorganisms9061310)
Supplement: Supplementary file 1 [file microorganisms-09-01310-s001.zip › microorganisms-1245046-supplementary.pdf]

Electronic supplemental material

Meropenem pharmacokinetics and target attainment in critically ill patients are not affected by extracorporeal membrane oxygenation: a matched cohort analysis

Matthias Gijsen [1,2*], Erwin Dreesen [1], Pieter Annaert [3,4], Johan Nicolai [3#], Yves Debaveye [5], Joost Wauters [6,7], and Isabel Spriet [1,2]

File S1

Definitions

Sepsis was defined as a documented or suspected infection complicated by at least two of the following criteria: leukocytosis (total white blood cell count >12x10^9/L), leucopenia (total white blood cells <4x10^9/L), increased C-Reactive protein plasma concentration (>15 mg/L), abnormal body temperature (<36 or >38.3°C), tachypnea (>20/min) or mechanical ventilation with adjusted respiratory rate, significant edema or a positive fluid balance (>20mL/kg/24h) and tachycardia (>90/min). Severe sepsis was defined as sepsis complicated with organ dysfunction. Organ dysfunction was defined by at least one of the following criteria: respiratory distress (PaO2/FiO2 <300), oliguria (urinary output <0.5 mL/kg.h for at least 2 hours with adequate fluid treatment (30 ml/kg crystalloid)), increased serum creatinine concentrations (>0.5 mg/dL relative to the baseline or >2 mg/dL), prolonged prothrombin time (INR >1.5), thrombocytopenia (platelet count <100x109/L), hyperbilirubinemia (total bilirubin concentration >4mg/dL) and elevated serum lactate concentration (>1.6 mmol/L). Septic shock was defined as severe sepsis complicated with hypotension despite adequate fluid resuscitation: systolic blood pressure <90 mmHg, a decrease in systolic blood pressure of >40 mmHg in relative to the baseline, diastolic blood pressure <70 mm Hg or need for vasopressor therapy [11, 12].

File S2

Bioanalytical method

Meropenem concentrations in plasma samples were quantified using a validated ultra-performance liquid chromatography – tandem mass spectrometry (LC-MS/MS) method.

Fifty microliter of each plasma sample was diluted with 500 µL of 0.1 M phosphate buffer containing cefoxitine (0.5 µg/mL). After adding 825 µL of acetonitrile, samples were vortexed for one minute to ensure protein precipitation. Subsequently, samples were centrifuged (20,816 g) for 10 min at 21°C. Following centrifugation, 800 µL of the supernatant was transferred into a stoppered glass test-tube (5 mL) containing 3 mL of dichloromethane. After vigorous vortexing (1 minute), the mixture was centrifuged (4,000 *g*) for 7 minutes at 21°C to separate the aqueous and organic layer. Next, 100µL of the aqueous top layer was diluted with 100 µL of water for injection. Hundred microliter of the resulting mixture was transferred into micro-inserts and sample vials before 10 µL was injected directly into the LC-MS/MS system.

Chromatographic separation was carried out on an Agilent 1200 HPLC-system. A kinetex® XB-C18 column (50 mm x 2.1 mm; 2.6 µm) was used for separation at a column temperature of 30°C. The mobile phase consisted of 0.1% formic acid in water for injection (A) and acetonitrile (B). Gradient elution at a constant flow rate (300 µL/min) was performed as depicted in Table S1.

Table S1. Liquid chromatography gradient used for separation of meropenem and cefoxitine.

Time (min)	A (%)	B (%)
0	96	4
0.5	96	4
0.51	80	20
1.50	80	20
1.51	70	30
3.00	70	30
3.01	96	4
7.00	96	4

A, 0.1% formic acid in H2O; B, acetonitrile;
gradient elution at a constant flow rate of 300 µL/min

Mass spectrometric analysis was performed using a triple quadrupole tandem mass spectrometer (API 2000 MS/MS, Applied Biosystems/MDS Sciex) equipped with a Turbo^{TM} ion source for electrospray ionization (ESI). ESI was operated at 400°C. The curtain gas was set at 30 psi, collision gas at 8 psi, and ion source gasses GS1 and GS2 at 50 and 40 psi, respectively. The components were detected by multiple reaction monitoring (MRM) and compound specific parameters for each m/z transition are shown in Table S2.

Table S2. Compound specific parameters.

	**Q1** (m/z)	**Q3** (m/z)	**Dwell time** (ms)	**DP** (V)	**FP** (V)	**EP** (V)	**CEP** (V)	**CE** (eV)	**CXP** (V)
Meropenem[a]	384.1	254.1	100	26	360	10.0	12	21	12
	384.1	297.9	100	26	360	10.0	18	21	14
	384.1	340.0	100	26	360	10.0	18	21	16
Cefoxitine[b]	425.9	156.0	150	-6	-330	-3.5	-20	-20	-10
	425.9	111.7	150	-6	-330	-3.5	-19	-20	-18

Q1: precursor ion; Q3: product ion; DP: declustering potential; FP: focusing potential; EP: entrance potential; CEP: cell entry potential; CE: collision energy; CXP: collision cell exit potential
[a] meropenem detected in positive ion mode with a retention time of 2.92 minutes; [b] internal standard, detected in negative ion mode with a retention time of 4.05 minutes

To determine concentrations of meropenem, a calibration curve was generated with spiked blank human plasma, which was prepared identically to the meropenem plasma samples. Linearity was obtained between 4.89 and 5000 ng/mL. Quality control samples (0.0858, 0.858, 8.58 µg/mL) were prepared in the same way. Quality control samples were used to assess within-run (repeatability) and between-day (reproducibility) precision. The calibration standards and quality control samples were prepared freshly each day to allow assessment of accuracy, linearity and reproducibility. The limit of quantification was defined as a proportion of signal-to-noise ratio of more than 10 and was at least 0.09 µg/mL. The limit of detection was defined as a proportion of signal-to-noise ratio of more than 3 and was at least 0.03 µg/mL.

File S3

NONMEM control stream

```
$PROBLEM    MEROPENEM POPPK
$INPUT      ID TIME DV EVID MDV AMT RATE CKDEPI BW
$DATA       DS.csv IGNORE=@
$SUBROUTINE ADVAN13 TOL=9
$MODEL      NCOMP=2 COMP=(CENTRAL,DEFDOS,DEFOBS) COMP=(PERI)
$PK
TVCL = THETA(1) *(BW/70)**THETA(5) *((CKDEPI/105)**THETA(9))

TVV1 = THETA(2) *(BW/70)**THETA(6)
TVV2 = THETA(3) *(BW/70)**THETA(7)
TVQ  = THETA(4) *(BW/70)**THETA(8)

CL   = TVCL*EXP(ETA(1))
V1   = TVV1*EXP(ETA(2))
V2   = TVV2*EXP(ETA(3))
Q    = TVQ *EXP(ETA(4))

S1   = V1
S2   = V2

K    = CL/V1
K12  =  Q/V1
K21  =  Q/V2

$DES
DADT(1) =  -K*A(1) -K12*A(1) +K21*A(2)
DADT(2) =           K12*A(1) -K21*A(2)

$ERROR
IPRED = F
IRES  = DV-IPRED
W     = SQRT(IPRED**2 * SIGMA(1,1) +SIGMA(2,2)) ; SD
IWRES = IRES/W
Y     = IPRED *(1+EPS(1)) +EPS(2)

$THETA
(0, 14.7)
(0, 25.5)
(0, 8.02)
(0, 5.51)
0.75 FIX
1.00 FIX
1.00 FIX
0.75 FIX
1.29

$OMEGA  BLOCK(2)
        0.219
        0.203 0.38
$OMEGA  0  FIX
$OMEGA  0  FIX

$SIGMA  0.083
        0  FIX
```

```
$ESTIMATION METHOD=1 INTERACTION MAXEVALS=9999 NSIG=3 SIGL=9 PRINT=5
NOABORT POSTHOC

$COVARIANCE PRINT=E UNCONDITIONAL
```

File S4

Details of ECMO treatment

All ECMO circuits were implanted surgically with peripheral cannulation. In both veno-venous (VV) and veno-arterial (VA) ECMO venous blood was removed from the vena femoralis, and passed through a circuit consisting of heparin-coated polyvinylchloride tubing (Medtronic Biomedicus Cannulae, Minneapolis, MN, USA), a centrifugal pump (Jostra Rotaflow, Maquet, Rastatt, Germany) or a diagonal pump (Medos Deltastream DP3, MEDOS Medizintechnik AG, Stolberg, Germany) generating the flow rate, a 1.9 m^2 polymethylpentene membrane oxygenator (Medos Hilite 7000 LT) and an integrated heat exchanger, prior to being returned to the body through a cannula to the upper part of the vena femoralis (VV) or to the arteria femoralis (VA). All ECMO circuits were primed with 600 mL of Plasmalyte solution (Baxter Healthcare Corp., Deerfield, IL).

Figure S1

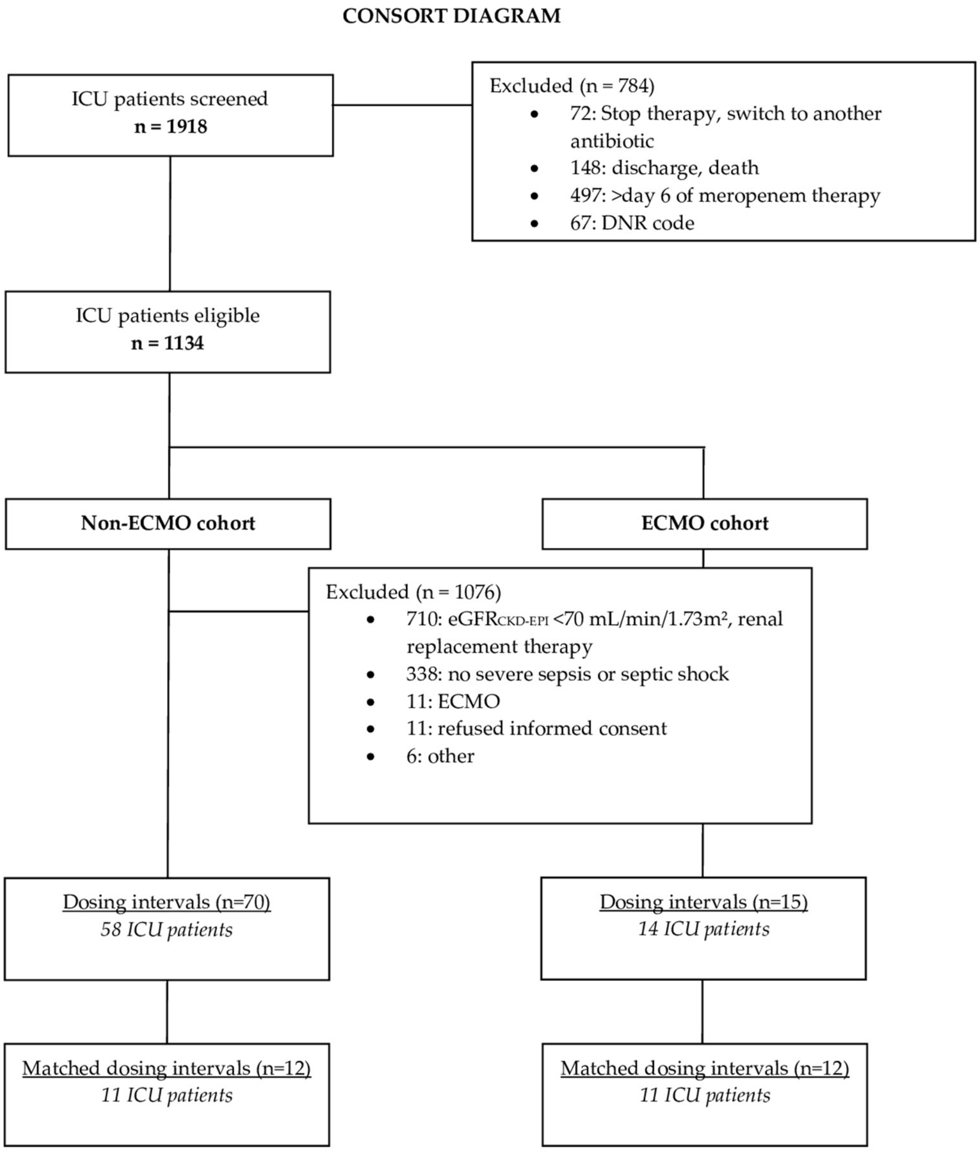

ICU = intensive care unit; DNR = do not resuscitate; ECMO = extracorporeal membrane oxygenation; $eGFR_{CKD-EPI}$ = estimated glomerular filtration rate according to the Chronic Kidney Disease Epidemiology Collaboration equation

Figure S1. Study flow diagram.

Figure S2

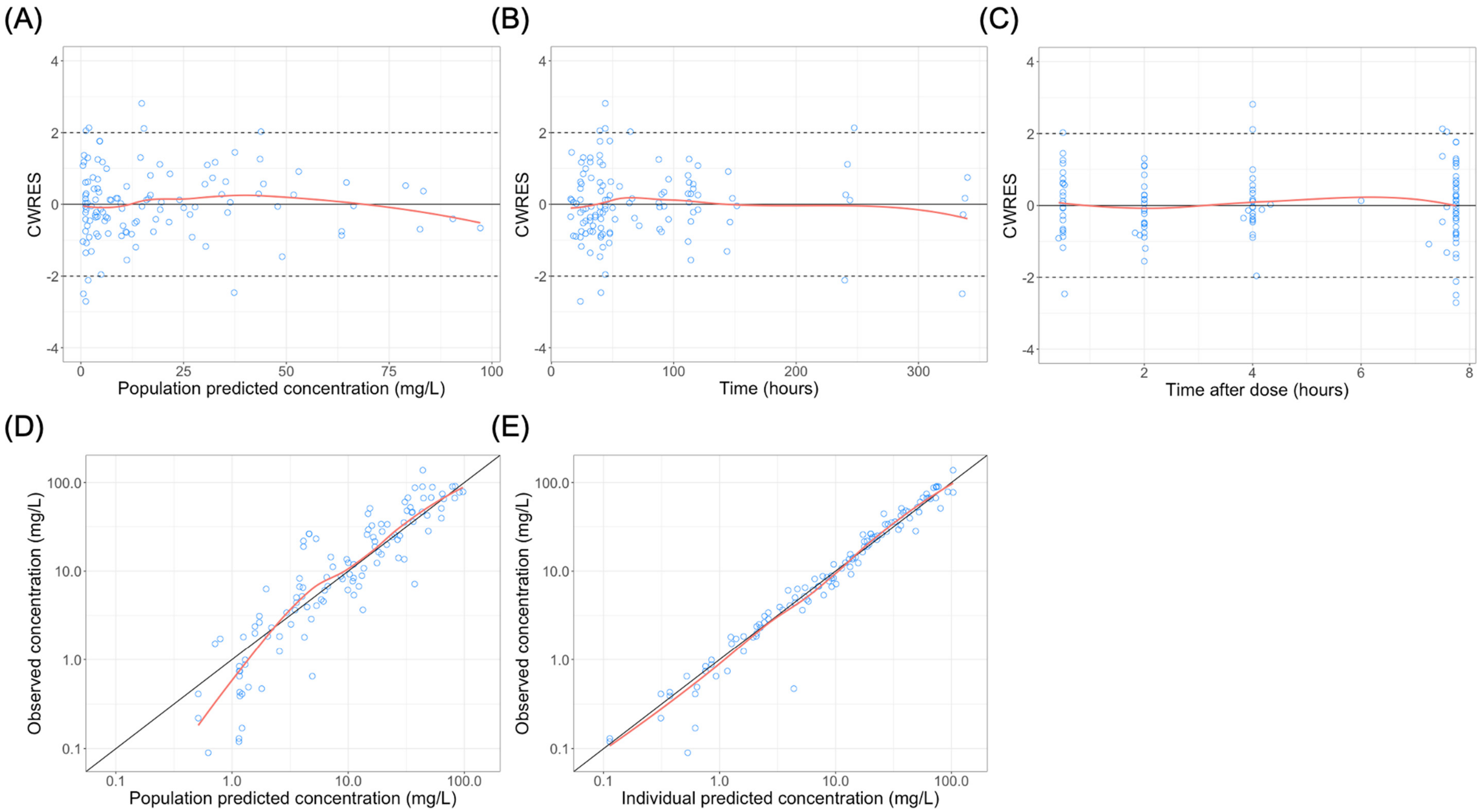

Figure S2. Goodness-of-fit plots of the final population pharmacokinetic model.

Figure S3

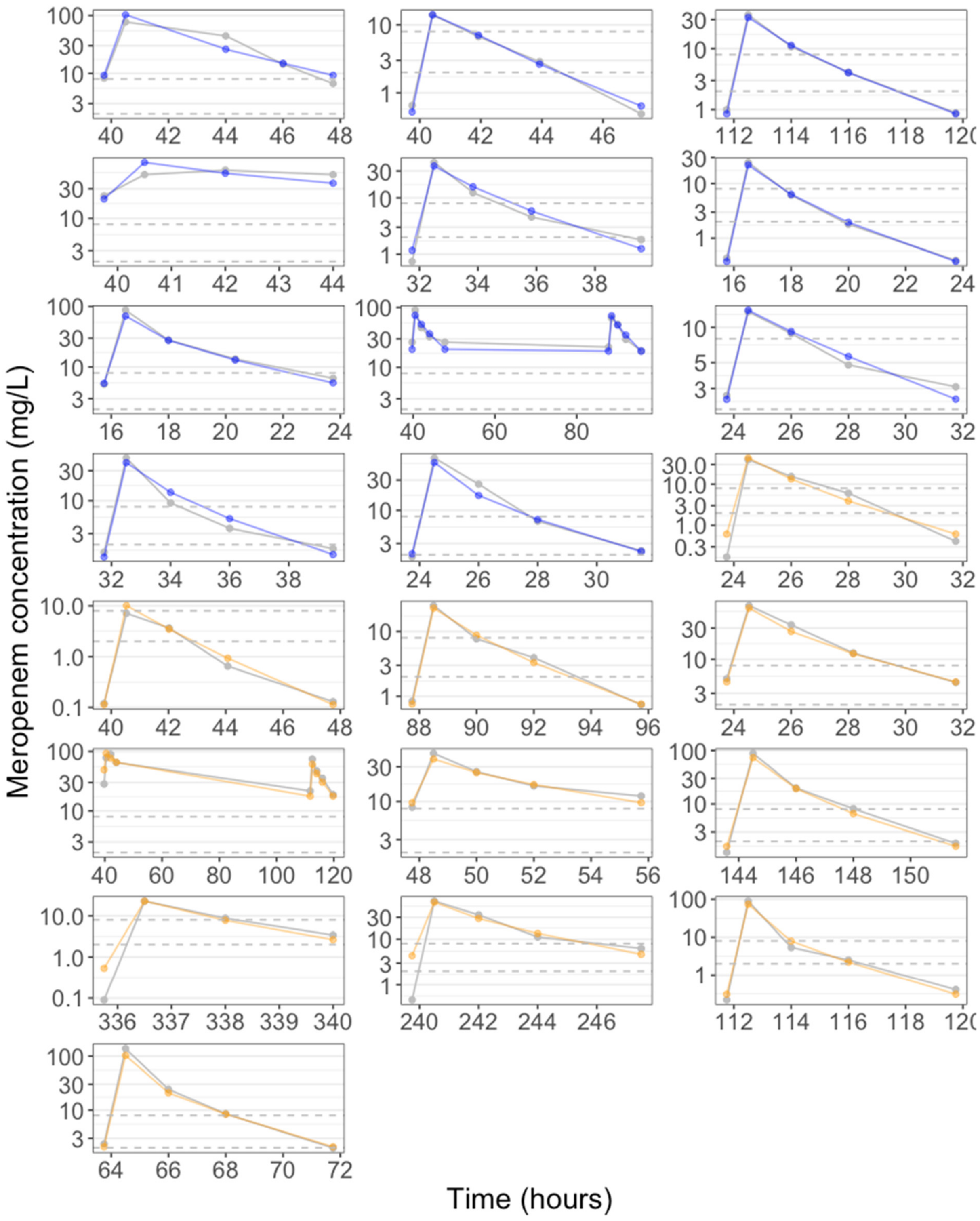

Figure S3. Individual pharmacokinetic profiles. Observed free meropenem plasma concentrations (grey) and predicted free meropenem plasma concentrations for non-ECMO (blue) and ECMO (orange) patients. PK/PD targets of 2 mg/L and 8 mg/L are indicated with dashed grey lines.
